# Blood donation for iron removal in individuals with HFE mutations: study of efficacy and safety and short review on hemochromatosis and blood donation

**DOI:** 10.3389/fmed.2024.1362941

**Published:** 2024-03-19

**Authors:** Laura Infanti, Gerda Leitner, Morten Moe, Vildana Pehlic, Marco Cattaneo, Pascal Benkert, Andreas Holbro, Jakob Passweg, Nina Worel, Andreas Buser

**Affiliations:** ^1^Regional Blood Transfusion Centre Swiss Red Cross Basel, Basel, Switzerland; ^2^Division of Hematology, University Hospital, University of Basel, Basel, Switzerland; ^3^Austrian Red Cross, Vienna, Austria; ^4^Unit of Medical Biochemistry, Division of Diagnostics and Technology, Akershus University Hospital, Akershus, Norway; ^5^Clinical Trial Unit, Department of Clinical Research, University and University Hospital Basel, Basel, Switzerland; ^6^Department for Transfusion Medicine and Cell Therapy, Medical University Vienna, Vienna, Austria

**Keywords:** iron removal, erythrapheresis, phlebotomy, HFE mutations, hemochromatosis, blood donation

## Abstract

**Background:**

Elevated serum ferritin with/without HFE variants in asymptomatic persons leads frequently to referral for blood donation. Hemochromatosis (p.C282Y/p.C282Y) only requires treatment. We evaluated safety and feasibility of iron removal in healthy persons with elevated ferritin and HFE variants using blood donation procedures.

**Materials and methods:**

Thirty subjects with ferritin >200 ng/mL (women) or >300 ng/mL (men) with p.C282Y/p.C282Y, p.C282Y/p.H63D or p.H63D/p.H63D were randomized to weekly phlebotomy (removal of 450 mL whole blood) or erythrapheresis (removal of 360 mL red blood cells) every 14 days. The ferritin target was <100 ng/mL. A full blood count and ferritin were measured at each visit. Hemoglobin (Hb) ≥140 g/L was required at inclusion. If Hb dropped to <120 g/L (women) or <130 g/L (men), procedures were postponed (7 or 14 days). Primary endpoint was the number of procedures needed to the ferritin target; secondary objectives were duration of treatment and compliance. The treatment effect was tested with Poisson regression; number of procedures and treatment duration were compared between study arms with the Kruskal–Wallis test.

**Results:**

Twenty-five of 30 participants were men (83%); mean age was 47 years (SD 10.5), mean BMI 26.6 kg/m^2^ (SD 3.6); 17 had p.C282Y/p.C282Y, nine p.C282Y/p.H63D, four p.H63D/p.H63D. Median baseline Hb was 150 g/L (IQR 144, 1,559), median ferritin 504 ng/mL (IQR 406,620). Twenty-seven subjects completed the study. Treatment arm (*p* < 0.001) and HFE variant (*p* = 0.007) influenced the primary endpoint significantly. To ferritin levels <100 ng/mL, a median number of 7.5 (IQR 6.2, 9.8) phlebotomies and 4.0 (IQR 3.0, 5.8) erythraphereses (*p* = 0.001) was needed during a median of 66.5 days (IQR 49,103) and 78.5 days (IQR 46139), respectively (*p* = 0.448). Low Hb was the principal reason for protocol violation; anemia occurred in 13 participants (48%). Immediate complications were infrequent; fatigue was reported after 25% of phlebotomies and 45% of erythraphereses. Thirty-five procedures were postponed because of low Hb and 15 for non-medical reasons. The median interval was 7.0 (IQR 7.7) and 14.0 (IQR 14, 20) days between phlebotomies and erythraphereses, respectively.

**Conclusion:**

Blood donation procedures remove iron effectively in HC, but frequent treatments cause Hb decrease and fatigue that can impair feasibility.

## Introduction

Clinicians frequently face a diagnostic and therapeutic dilemma when incidentally detecting elevated serum ferritin as an isolated finding or along with a mutation of the HFE gene in patients seeking medical care for other reasons. Because many of these patients have no associated major clinical conditions and no symptoms of iron overload, a frequent measure is to recommend “therapeutic blood donation.” However, the only well-defined indication for iron removal is hemochromatosis (HC), a diagnosis that is not always easily formulated by both primary care providers and physicians taking care of blood donors. The BioIron group of experts recently provided a clear definition of HC: a genetic condition impairing the hepcidin-ferroportin axis that is caused by the homozygous HFE p.C282Y variant and is associated with elevated transferrin saturation, iron depositions in the liver and clinical manifestations of iron overload in the absence of a red blood disorder (1). Based on the available evidence, the expert group also stated that the p.C282Y/p.H63D HFE genotype should not be considered diagnostic for HC but rather as an additional factor for iron accumulation if other risks for liver disease are present (2, 3), and underlined that a homozygous p.H63D and any heterozygous HFE variant do not predispose to clinically relevant iron overload (4). The less frequent p.S65C variant has a similar effect on iron accumulation as the p.H63D (5).

Conventional phlebotomy is the standard therapeutic approach for HC. Initial treatment consists in venesection at weekly intervals to rapidly reduce iron stores (induction phase), followed by a maintenance phase with regular phlebotomy at individual intervals so as to avoid iron reaccumulation. Clinical guidelines recommend to initiate treatment in all p.C282Y homozygotes with serum ferritin above the upper reference limit of 300 ng/mL in men and 200 ng/mL in women, while the ferritin level to be targeted with the induction phase is indicated to be below 100 ng/mL (6, 7) or less than 50 ng/mL (8). Ferritin values near 50 ng/mL or below 100 ng/mL are recommended for the maintenance phase.

Studies conducted in HC patients with highly elevated ferritin and clinical manifestations of iron overload demonstrated that red blood cell (RBC) apheresis is more efficacious for rapidly removing iron compared to conventional phlebotomy (9–12), so that this method was included in the ASFA Guidelines as a treatment for HC in 2013 (category I, evidence 1B) (13).

Both phlebotomy and RBC erythrapheresis, which allows the collection of single- or double-units of RBC, are established procedures for blood donation. However, whether blood centers should be allowed to treat persons with HC and to use blood products for transfusion is still a matter of discussion. Currently, the acceptance of persons with HC as blood donors is handled variably by national and local regulations (14), although both the Council of Europe (15) and the FDA (https://www.accessdata.fda.gov/scripts/cdrh/cfdocs/cfcfr/CDFSearch.cfm, accessed on 12/28/2023) approve blood collection for transfusion purposes from HC subjects in defined circumstances. Moreover, in countries where blood donation of HC carriers is permitted, the clearance of blood products is allowed either regardless the ferritin level of the donor or after ferritin is lowered within the normal range by previous iron depletion. As to date, uncertainty regarding these issues is due principally to the lack of robust data endorsing or confuting a safe usage of blood components derived from HC subjects, although no safety concerns have emerged in centers where blood of HC donors has been transfused for years (16, 17).

In 2004, we implemented both routine ferritin measurement in blood donors and double-dose erythrapheresis (erythrapheresis) at our institution. In the same year, new Swiss regulations were elaborated stating that blood of HC persons can be cleared for transfusion provided that the donor’s ferritin level is within normal ranges and the donation frequency does not exceed that defined for all blood donors (a maximum of three times/year for women and four times/year for men). Accordingly, blood drawn from persons with HC, even if fulfilling the eligibility criteria for blood donation, is discarded as long as ferritin is elevated.

A first evaluation of our experience with 86 carriers of HFE variants donating whole blood or RBC units with erythrapheresis (18) prompted the initiation of a randomized study on iron removal in persons with HC using routine blood donation procedures. The study was conducted at the centers of Basel and Vienna and aimed to explore the feasibility of frequent procedures as well as patterns and changes of iron indices in otherwise healthy persons with different HFE genotypes (reported in (19)). In designing the study, an intensive treatment frequency was intentionally defined. This choice mirrors the aggressive approach that is often adopted by many clinicians in treating preclinical HC, as observed in our experience.

Hereby, we present the results of efficacy and feasibility of phlebotomy and erythrapheresis performed as in the routine of the blood donation center in treating individuals who carry HC and other HFE genotypes, and who potentially qualify as blood donors. The short review of the current practices on HC and blood donation aims to provide practical information to clinicians who consider this therapeutic option for their patients with elevated ferritin and HFE variants.

## Methods

### Screening and recruitment of the study subjects

Potential study participants were evaluated at the sites of Basel and Vienna either by screening of first-time blood donors or upon referral from primary care practitioners. Eligible for the study were persons with no relevant clinical condition aged 18 to 65 years with the following: p.C282Y, p.H63D, or p.S65C HFE variants in homozygous or compound heterozygous combination; elevated ferritin (above 200 ng/mL in females, above 300 ng/mL in males); a total blood volume of at least five L (based on sex, height and weight) and hemoglobin (Hb) of 140 g/L or greater as required for erythrapheresis. At inclusion, information on relevant associated medical conditions and ongoing medication was collected and the body mass index (BMI) was determined.

Eligibility for both the study and for blood donation was assessed by a physician with an interview and using the standard blood donor questionnaire, and based on the screening laboratory tests (described below) as well. Participants were randomized 1:1 to phlebotomy or erythrapheresis by the Clinical Trial Unit of the University of Basel using a mixed randomization scheme (20).

### Study procedures, treatment schedule, and handling of adverse events

Phlebotomies and erythraphereses were performed following internal standard procedures and complying with national directives. With phlebotomies, 450 mL whole blood was drawn corresponding to the removal of 200–250 mg iron. With erythapheresis, a fixed volume of 360 mL RBC corresponding to 360–370 mg iron was collected with the ALYX device (Fenwal-Baxter, Zürich, Switzerland). Phlebotomies were scheduled weekly and erythaphereses every 14 days until ferritin was below 100 ng/mL. A 2- or a 4 days delay from a scheduled visit was permitted for the participants undergoing phlebotomy and erythrapheresis, respectively.

The European regulations define a minimum Hb of 125 g/L for women and 135 g/L for men for whole blood donation, and of 140 g/L for both sexes for double-dose erythrapheresis (15). For the inclusion in the study, a minimum Hb value of 140 g/L was required regardless of sex and treatment arm. If Hb dropped below 140 g/L during treatment, a study physician had to decide whether a phlebotomy or erythrapheresis could be performed. In case of anemia (Hb below 120 g/L in women and below 130 g/L in men), phlebotomies were postponed by seven days and erythraphereses by 14 days.

Immediate adverse events (AEs) of procedures were recorded at the study site and delayed complications were reported by the participants on a questionnaire after each visit. AEs were assessed for severity and imputability by a physician according to national standards. Other reasons for missing or delaying a scheduled appointment were also recorded.

The study ended with a follow-up visit 8 weeks after the target ferritin value was achieved. After completing the study, the participants had the possibility to undergo further treatment with regular phlebotomy or erythrapheresis at appropriate intervals regardless of their eligibility for blood donation.

### Laboratory assessment

At each study visit, laboratory assessment included a complete blood count (Sysmex K-4500, Sysmex Digitana AG, Horgen, Switzerland) on a venous blood sample and ferritin determination. Beside ferritin, several other iron indices were analyzed for a separate evaluation of some secondary outcomes (as reported in (19)). Infectious disease markers were tested as for routine blood donation (HBsAg, HIV p24/antibodies, HCV, and syphilis antibodies; Architect ci8200, Abbott Diagnostics, Abbott Park, IL, United States) including NAT for HBV, HCV and HIV when the blood drown from the study participants could be used for transfusion, as explained below.

### Manufacturing and clearance of blood products

In Basel, RBC and plasma products obtained from phlebotomies and RBC units collected with erythrapheresis were issued for transfusion as soon as ferritin was within normal limits, provided that eligibility requirements were fulfilled, and infectious disease markers were negative. Since the Austrian regulations do not allow blood donation in subjects with HC, blood from the study participants recruited in Vienna was not transfused.

### Statistical analysis

The primary endpoint of the study (efficacy) was the number of phlebotomies and erythraphereses needed to reach a ferritin value below 100 ng/mL.

Secondary objectives included the number of days needed to reach the ferritin target and the evaluation of safety and feasibility: immediate and delayed AEs, occurrence of anemia; number of procedures postponed or not performed for any reason. Outcomes of iron biomarkers are described in Infanti et al. (19). In addition, and limited to the site of Basel, the number of blood components used for transfusion was evaluated.

Based on the study by Rombout 2012 (9), a total sample size of 30 subjects was calculated to be necessary to ensure 22 evaluable participants in order to address the primary and secondary objectives and anticipating a drop-out rate of 25%. The resulting power for showing a difference in the primary endpoint between the two treatment arms would be 95%, when the mean number of required erythraphereses were half the one of phlebotomies.

The treatment effect on the primary endpoint was tested by Poisson regression, with sex, HFE variant, BMI and baseline ferritin as covariates. In a successive additional analysis, age, blood volume and number of previous donations were also tested as covariates. The Kruskal–Wallis test was used to compare the number of procedures and the treatment duration between the two study arms (*p*-value <0.05 considered as statistically significant). Demographics, relevant baseline variables, AEs and other results of the secondary endpoints were summarized with descriptive statistics. Categorical and numerical data are presented as frequencies and percentages, median and interquartile range (IQR) or average and standard deviation (SD), as appropriate. All analyses were conducted using the statistical software package R, version 4.3.2.

The trial was registered in Basel (Trial Number USB-2013-059). Ethical approval was obtained for both the study site of Basel (Ref. EK 94/13) and Vienna (Ref. EK 1015/2015). All participants gave their written consent. The study was supported by the research fund of the Blood Transfusion Centre Swiss Red Cross Basel.

## Results

### Recruitment and characteristics of the study participants

**Figure 1:**
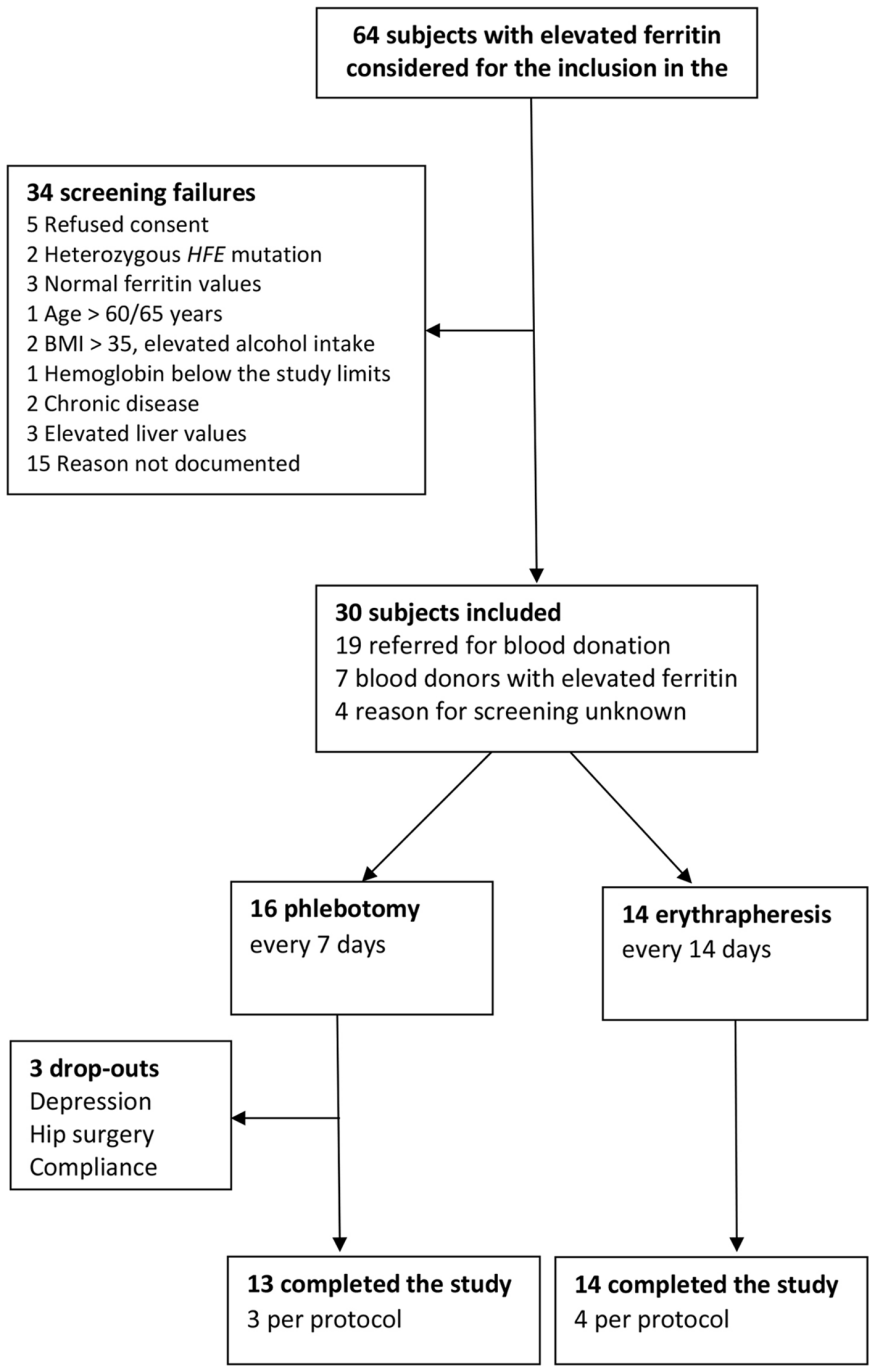
Consort diagram.

The study started in October 2013 (enrolment of subject 1) and was completed in October 2017 (follow-up visit of subject 30). The consort diagram in Figure 1 provides an overview of the study course and Table 1 summarizes the characteristics of the study participants.

**Table 1:** Clinical characteristics and laboratory values of the study participants.

	Phlebotomy Subjects *n* = 16	Erythrapheresis Subjects *n* = 14	All subjects *n* = 30
Men	15	10	25 (83%)
Age (years)	48 (12)	46 (8.5)	47 (10.5)
HFE mutation			
p.C282Y/p.C282Y	8	9	17
p.C282Y/p.H63D^a^	5	4	9
p.H63D/p.H63D^b^	1	3	4
Blood donation			
With previous blood donation	4	3	7
No. previous blood donations	3 (2, 6)	1 (1, 5)	3 (1, 6)
Eligible	13	10	23 (75%)
Not eligible, reasons:	3	4	7
National regulations	2	2	4
Previous malignancy		2	2
Risk for vCJD	1		1
Clinical characteristics			
Body-mass index (kg/m^2^)	26.6 (4.1)	26.6 (3.0)	26.6 (3.6)
Associated medical conditions			
Hypertension	4	4	8
Subclinical hypothyroidism	3		3
Depression		2	2
Asthma	1		1
Vitamin B12 deficiency	1		1
Laboratory values at baseline			
Hemoglobin (g/L)	147 (145, 154)	151 (141, 155)	150 (144, 155)
Ferritin (ng/mL)^a^	521 (447, 633)	476 (356, 610)	504 (406, 620)

Values are mean (SD), median (range), or median (IQR).

a One man with p.C282Y/p.S65C.

b One man with p.H63D/p.S65C.

Sixty-four subjects were screened because of elevated ferritin and 30 (47%) were found to carry a p.C282Y or p.H63D homozygous HFE variant or a p.C282/p.H63D genotype and were enrolled in the study (26 in Basel and four in Vienna). Of these, 19 were screened upon referral for blood donation, seven were blood donors with an incidental finding of elevated ferritin, and four were screened for unknown reasons. There were 25 men (83%); mean age was 47 years (SD 10.5) and mean BMI was 26.6 kg/m^2^ (SD 3.6). The HFE genotype was p.C282Y/p.C282Y in 17 subjects (four women), p.C282Y/p.H63D in nine (one woman, one men with p.C282Y/p.S65C), and p.H63D/p.H63D in four men (one with p.H63D/p.S65C). Of the seven blood donors, none had iron depletion treatment previously. The median number of previous blood donations was of 3 (range: 1–6). Twenty-three of the 30 enrolled subjects (75%) fulfilled the eligibility criteria for blood donation; ineligible were the four participants with HC recruited in Vienna, two women previously treated for breast cancer and melanoma, and one man with risk for variant CJD. The most frequent comorbidities requiring medication were hypertension (eight subjects) and subclinical hypothyroidism (three). At enrolment, median Hb was 150 g/L (IQR 144, 155) and median ferritin 504 ng/mL (IQR 406, 620).

### Efficacy of iron depletion treatment

**Figure 2:**
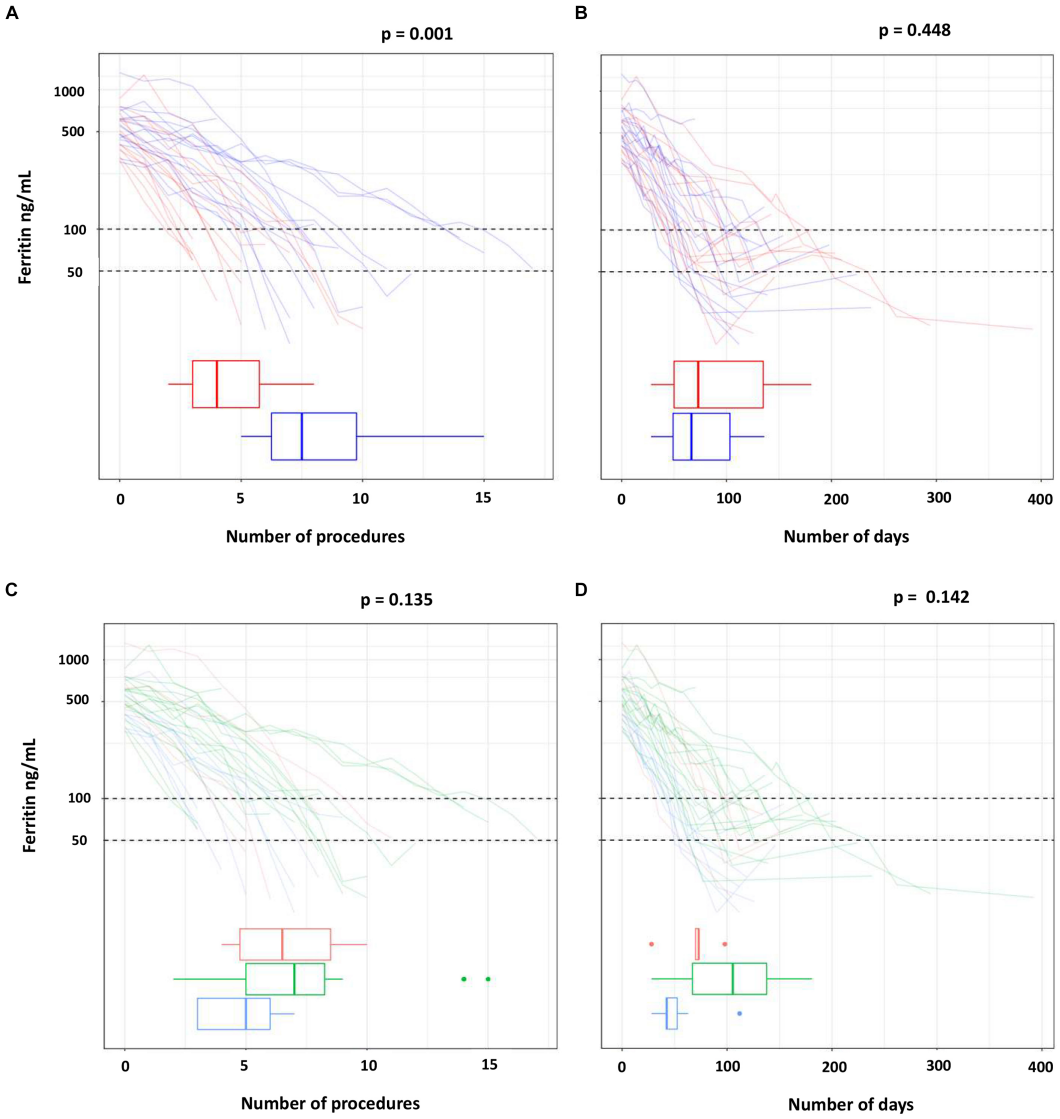
Number of procedures and of days to reach the target ferritin value by treatment **(A,B)** and by HFE mutations **(C,D)**.

Table 2 and Figure 2 show the number of procedures and the days needed to reach ferritin levels below 100 ng/mL in each study arm (Figures 2A,B) and in the HFE genotype groups (Figures 2C,D). Data are also reported for the more restrictive ferritin target of 50 ng/mL or less.

**Table 2:** Number of procedures and of days needed to ferritin target by treatment and by HFE genotype (*p*-values from Kruskal–Wallis tests).

By treatment^a^	Phlebotomy subjects *n* = 16	Erythrapheresis subjects *n* = 14		*p* value
Ferritin < 100 ng/mL				
Subjects with ferritin <100 ng/mL	14 (88%)^b^	14 (100%)		
Procedures, median (IQR)	7.5 (6.2, 9.8)	4.0 (3.0, 5.8)		*p* = 0.001
Days, median (IQR)	59.0 (49.0, 103.2)	78.5 (46.0, 139.0)		*p* = 0.448
Ferritin < 50 ng/mL				
Subjects with ferritin <50 ng/mL	8 (50%)	8 (57%)		
Procedures, median (IQR)	8.0 (6.0, 9.5)	5.0 (3.8, 5.8)		*p* = 0.017
Days, median (IQR)	66.5 (42.8, 70.0)	99.5 (66.5, 183.8)		*p* = 0.092
By HFE mutation	p.C282Y/p.C282Y subjects *n* = 17	p.C282Y/p.H63D subjects *n* = 9	p.H63D/p.H63D subjects *n* = 4	
Ferritin < 100 ng/mL				
Subjects with ferritin <100 ng/mL	16 (94%)	8 (89%)	4 (100%)	
Procedures, median (IQR)	7.0 (5.0, 8.2)	5.0 (3.0, 6.0)	6.5 (4.8, 8.5)	*p* = 0.135
Days, median (IQR)	105.5 (67.0, 137.8)	42.5 (42.0, 52.5)	71.5 (59.5, 79.2)	*p* = 0.015
Ferritin < 50 ng/mL				
Subjects with ferritin <50 ng/mL	6 (35%)	7 (78%)	3 (75%)	
Procedures, median (IQR)	8.5 (8.0, 9.0)	5.0 (3.5, 6.0)	6.0 (5.5, 8.5)	*p* = 0.058
Days, median (IQR)	133.0 (69.2, 201.2)	56.0 (42.5, 66.5)	87.0 (61.0, 124.0)	*p* = 0.142

a HFE genotype: p.C282Y/p.H63D in two subjects (one in each arm), p.C282Y/p.C282Y in 12 subjects.

b Two drop-outs; the third drop-out reached the ferritin target.

The ferritin target was not achieved in two of the three drop-outs of the phlebotomy arm (one man with p.C282Y/p.C282Y, one man with p.C282Y/p.H63D). Ferritin dropped below 50 ng/mL in eight participants in each treatment arm, but less frequently in the p.C282Y homozygotes (in six of 17 subjects). The median number of phlebotomies required to lower ferritin below 100 ng/mL was 1.9 times that of erythraphereses (7.5 [IQR 6.2, 9.8] vs. 4.0 [IQR 3.0, 5.8]; *p* = 0.001). This result was confirmed by the adjusted regression model reported in Table 3, in which the mean number of phlebotomies is 1.8 times that of erythraphereses (95% CI [1.3, 2.6]; *p* < 0.001). The median duration of treatment was 59.0 days (IQR 49, 103) and 78.5 days (IQR 46.0, 139.0) (*p* = 0.448), respectively. For achieving a ferritin value of 50 ng/mL or less, one more procedure in each arm was necessary, about 7 days with phlebotomy but more than 20 additional days if using erythrapheresis.

**Table 3:** Effect of each covariate on the primary endpoint (number of procedures to ferritin <100 ng/mL): likelihood-ratio test and estimated multiplicative effects (Poisson regression model).

Variable	Estimate	95% CI	*p*-value
Sex: male vs. female	1.292	0.813, 2.144	0.288
BMI for each additional kg/m^2^	1.011	0.970, 1.054	0.607
Ferritin for each additional ng/mL	1.001	1.000, 1.001	0.226
HFE p.C282Y/p.H63D vs. homozygous p.C282Y	0.624	0.427, 0.897	0.011 (0.007 for the whole variable)
HFE homozygous p.H63D vs. homozygous p.C282Y	0.616	0.371, 0.981	0.041 (0.007 for the whole variable)
Phlebotomy vs. double-dose erythrapheresis	1.844	1.340, 2.568	<0.001

The number of procedures needed to ferritin below 100 ng/mL was highest for the p.C282Y homozygotes (median 7.0; IQR 5.0, 8.2) and lowest for the subjects with p.C282Y/p.H63D (median 5.0; IQR 3.0, 6.0). Also, the number of days needed to reach the ferritin target was highest for the p.C282Y/p.C282Y group (median 105.5; IQR 67.0, 137.8), followed by the p.H63D/p.H63D (median 71.5; IQR 59.5, 79.2) and by the p.C282Y/p.H63D (median 42.5; IQR 42.0, 52.5).

Overall, treatment arm and HFE variant were the covariates with a significant effect on the primary endpoint (*p* = 0.001 and *p* = 0.007 in multivariate Poisson regression, respectively) (Table 3). However, there were three p.C282Y homozygotes treated with phlebotomy who required a greater number of procedures (14 to 15) for reaching the ferritin target. After excluding these individuals, in the multivariate Poisson regression the effect of treatment on the primary endpoint was still statistically significant (*p* = 0.011), but that of the HFE variant was not (*p* = 0.311) (data not shown). Age, blood volume and number of previous blood donations included as additional covariates had no relevant effect on the result, in particular there was no change on the confidence interval (data not shown).

### Safety and feasibility of treatment

Table 4 summarizes the data of feasibility of the study procedures, including protocol violations, AEs, and the blood usage of blood collections.

**Table 4:** Study visits, study procedures, and blood products collected.

Per treatment arm	Phlebotomy subjects *n* = 16	Erythrapheresis subjects *n* = 14	All subjects *n* = 30	
Study visits				
All study visits	149	71	220	
Laboratory check only^a^	16	20	36	
Participants completing treatment, *n*				
Total	13	14	27	(90%)
Per protocol	3	4	7	(26%)
With Hb < 140 g/L^b^	10	10	20	(74%)
With Hb < limits of anemia^c^	8 (7 men, 1 woman) (50%)	5 (1 man, 4 women) (36%)	13	(48%)
Procedures, *n*				
Completed	147 (99%)	66 (93%)	213	(97%)
With Hb < 140 g/L^b^	28 (19%)	17 (24%)	45	(20%)
With Hb < limits of anemia^c^	5	3	8	(4%)
Interrupted	2 (1 no flow, 1 technical issue)	5 (3 hematoma, 1 no flow, 1 technical issue)	7	(3%)
Not performed, low Hb	12 (8%)	9 (13%)	21	(10%)
Not performed, other reasons	5	1	6	(3%)
Postponed, low Hb	16	19	35	(13%)
Postponed, other reasons	8	6	14	(7%)
Interval, days (median, IQR)	7 (7, 7)	14 (14, 20)		
Adverse events, *n*				
Local hematoma	13	14	27	(13%)
Fatigue following procedure	37 (25%)	31 (47%)	68	(32%)
Blood products				
Blood collections discarded^d^	107 (72%)	47 (66%)	154	(70%)
Blood collections processed	42	24	66	
Red blood cell/plasma units used	42/42	48/−^e^	90/42	
Total blood components used	84	48	132	

Per HFE genotype	Homozygous p.C282Y subjects *n* = 17	Other genotypes subjects *n* = 13	All subjects *n* = 30	
Study visits				
All study visits	144	76	220	
Laboratory check only^a^	19	17	36	
Participants completing treatment				
Total	15 (88%)	12 (92%)	27	(90%)
Per protocol	6	1	7	(26%)
With Hb < 140 g/L^b^	9	11	20	(74%)
With Hb < limits of anemia^c^	5 (2 men, 3 women) (29%)	8 (6 men, 2 women) (61%)	13	(48%)
Procedures, *n* (%)				
Completed	132	81	213	(97%)
With Hb < 140 g/L^b^	27	18	45	(20%)
With Hb < limits of anemia^c^	8	0	8	(4%)
Interrupted	4 (1 hematoma, 1 technical problem, 2 no flow)	3 (2 hematoma, 1 technical problem)	7	(3%)
Not performed, low Hb	13	8	21	(10%)
Not performed, other reasons	5	1	6	(3%)
Postponed, low Hb	22	13	35	(13%)
Postponed, other reasons	8	6	14	(7%)
Interval, days (median, IQR)	14 (7,14)	14 (7,14)		
Adverse events, *n* (%)				
Local hematoma	16	9	27	(13%)
Fatigue following procedure	54 (41)	14 (17)	68	(32%)
Blood products				
Blood collections discarded^d^	106	48	154	(70%)
Blood collections processed			66	
Red blood cell/plasma units used	51/15	39/27	90/42	
Total blood components used	66	66	132	

a Including visits with no procedure performed, visits for hemoglobin and ferritin determination only, and follow-up.

b Protocol deviation.

c Hemoglobin below 120 g/L for women, 130 g/L for men.

d Because of ferritin above the normal limits or because of permanent or temporary ineligibility for blood donation (excluding interrupted procedures).

e Two red blood cell units from each apheresis, no plasma collection.

During the study, a total of 256 visits were performed, including 149 for phlebotomy, 71 for erythrapheresis and the remaining for laboratory assessment only.

In total, 27 participants completed the study (90%), but of these only seven (26%) were treated per protocol. The reason for a protocol violation was almost exclusively low Hb. In the phlebotomy arm, only three participants had Hb values of 140 g/L or more at all study visits; in 10 subjects, phlebotomies were performed despite lower levels and in eight (50%, 7 men, 1 woman) Hb dropped below the limits of anemia. All 14 subjects in the erythrapheresis arm completed the study, of these four per protocol. In 10 subjects, at least one apheresis was made despite Hb values lower than 140 g/L; anemia occurred in five participants (36%, 1 man, 4 women). In total, 13 anemia occurred in 13 participants (48%). Comparing the HFE genotype groups, the number of p.C282Y homozygotes completing the study per protocol was higher, and the proportion of those developing anemia was lower (29% vs. 61% of subjects with other HFE variants).

Of the procedures, 19% phlebotomies and 24% of erythraphereses were done despite Hb lower than 140 g/L; 35 (13%, 16 phlebotomies and 19 erythraphereses) were postponed because of low Hb and 15 (6%) for non-medical reasons. Two (1%) phlebotomies and five (7%) erythraphereses were interrupted because of technical problems or local hematoma. Local hematoma was more frequent with erythrapheresis (in 21% of the procedures) but caused an interruption in three cases only. Fatigue was the most frequent AEs and was reported after 25% of phlebotomies and 47% of erythraphereses and was more frequently reported by participants with p.C282Y/p.C282Y (41% vs. 17% of individuals with other HFE genotypes). Despite low Hb and AES, the median interval between procedures was not longer than that defined in the study protocol, was 7 days (IQR 7, 7) between phlebotomies and 14 days (IQR 14, 20) between erythraphereses, and comparable between participants with HC and with other HFE variants.

In Basel, 94 RBC concentrates and 56 plasma units obtained from the study participants were cleared for transfusion.

## Discussion

Based on previous observational data from our institution (18), we conducted a randomized study on whole blood phlebotomy and double-dose erythapheresis, two routine modalities of blood donation, for iron depletion in physically fit individuals with preclinical HC and other HFE variants. Confirming previous experiences in symptomatic HC patients (11, 12, 21), an effective ferritin reduction was achieved with both methods with very few immediate AEs, whereas erythrapheresis removed iron more quickly compared to phlebotomy. However, decreasing Hb levels and fatigue were the most relevant consequences of frequent treatments with both types of procedure.

The study population comprised almost exclusively middle-aged men with moderately elevated ferritin and adequate Hb levels, reflecting the typical characteristics of patients referred to a blood donation center for iron removal. Because one objective of the study was evaluating iron indices in different HFE genotypes during treatment, enrolled were not only subjects with HC but also individuals with p.C282Y/p.H63D and p.H63D/p.H63D. In the publication describing these results, non-transferrin bound iron, the cytotoxic iron form, was detected in all subjects with p.C282Y/p.C282Y and in only few of those with p.C282Y/p.H63D, corroborating current guidelines that recommend treatment in all persons with HC having increased ferritin but not in all individuals with the p.C282Y/p.H63D genotype (19). It is a common experience, however, that clinicians often prescribe iron removal also for patient with elevated ferritin for whom treatment is not actually indicated, such as in those with the homozygous p.H63D HFE variant. A survey among Norwegian blood centers revealed a diffuse uncertainty among both primary care physicians and transfusion medicine specialists in prescribing and performing iron depletion treatment. One relevant difficulty for decision making was attributed to the confusing terminology in common use (e.g., “clinical HC,” “preclinical hemochromatosis,” “biochemical hemochromatosis” “risk person for developing iron overload”) (22). Therefore, it is important to emphasize the relevance of both an accurate diagnosis of HC and an appropriate indication for iron removal, because frequent blood drawing is associated with a risk of anemia and other potentially harmful side effects.

In the present study, the factors with a relevant impact on the primary endpoint were treatment arm and HFE genotype. The ferritin target was reached with a lower number of erythraphereses (a median of 4 vs. 7.5 phlebotomies) compared to phlebotomy, although with a longer treatment time (a median of 78.5 vs. 59 days for phlebotomy). The longer treatment duration can be principally explained by the fact that34% of the procedures had to be postponed. Similarly, in the first randomized study on phlebotomy and erythrapheresis in symptomatic HC patients (9), the number of erythraphereses needed to achieve a ferritin below 50 ng/mL was 70% lower compared to that of phlebotomies. Three p.C282Y homozygotes of the phlebotomy arm required more procedures to complete the treatment. These subjects did not display any relevant differences in their clinical or laboratory features compared to the other study participants. Excluding these individuals, the Poisson regression confirmed the statistically significant effect of treatment type on the primary endpoint, but not that of the HFE genotype, a result that is possibly explained with the small number of subjects included in the analysis. Also, it is a common observation in clinical practice that the duration of treatment and the number of venesections needed for iron depletion in HC can vary considerably even among individuals with similar clinical characteristics and baseline laboratory values. Other factors such as age, blood volume and number of previous blood donations did not have an impact on the primary endpoint, but again no further conclusion can be drawn on this point due to the limited number of subjects tested.

In that trial, immediate AEs (vaso-vagal reactions, dizziness, mild citrate toxicity) occurred infrequently with both methods. Another randomized study with a design similar to that of our trial (removal of 450 mL whole blood weekly and of 400 mL RBC every two weeks) showed a more rapid decrease of ferritin with erythrapheresis to the target value with a comparable median treatment duration in the two arms. The main differences of the present study to those previous works are that in our study asymptomatic persons only were included, the iron removing procedures were performed following standard modalities and were not adapted to the subjects’ characteristics (e.g., body weight), and both immediate and delayed AEs of treatment were recorded, allowing a more accurate evaluation of tolerability. In particular, fatigue, that was reported by the participants after 32% of the treatments (more often after erythrapheresis and by subjects with HC), may represent a more relevant AE than usually appreciated. Fatigue following bloodletting may negatively affect quality of life, and impair adherence to treatment in HC patients and return to blood donation in healthy volunteers (23), as shown in a cohort of blood donors undergoing regular double-dose eyrthrapheresis (24).

Considering all the aspects discussed above and the elevated proportion of participants who developed anemia in our study (29%), for asymptomatic persons with HC who are mostly healthy and have a moderate ferritin elevation, we think that the goal of clinical management should not be necessarily a rapid, aggressive lowering of ferritin but much more the long-term binding to regular bloodletting or blood donation. Moreover, the frequent occurrence of fatigue in subjects with HC observed in our study reinforces the necessity of a personalized approach that should not be merely based not merely on ferritin values. Because today HC is diagnosed in a preclinical phase in the majority of cases, offering blood donation instead of discarding the blood drawn can represent an important motivation that is likely to ameliorate compliance. The most recent guidelines of the European Association for the Study of the Liver indicate personalized erythrapheresis as the preferred treatment in selected patients with HC and clinical manifestations of iron overload and suggest regular blood donation for individuals with at risk genotypes and increased transferrin saturation, but with normal ferritin levels, particularly if identified in early adulthood (8). However, iron removal for HC is not possible in every blood donation facility, as discussed in the short review below.

Strengths of the present study is that we evaluated a well characterized population with homogeneous clinical and laboratory characteristics and conducted a direct comparison between a group of individuals with HC and subjects with other HFE genotypes. The main limitation is represented by the small number of participants included in total and in each study arm, although the sample size was adequate to allow robust statistical conclusions on the primary endpoint. In addition, our analysis. Moreover, our analysis was short termed focusing on the period of treatment only.

The issue of iron depletion in preclinical HC and in different situations of elevated ferritin deserves further evaluation as it represents a frequent clinical situation and because treatment can be further optimized. Topics of interest for future research include the comparison of different frequencies of phlebotomy and erythrapheresis, the assessment of the role of iron removal in persons with HFE genotypes other than the p.C282Y/p.C282Y, longitudinal evaluations of blood donors with preclinical HC, and the elaboration of strategies to enhance adherence to long-term bloodletting or blood donation.

In conclusion, our study shows that a tight schedule of iron removal with blood donation procedures is feasible and safe, but it is associated with side effects that are potentially relevant even in subjects with no major clinical conditions and normal Hb levels and that can impair compliance to treatment. Therefore, iron removal must be reserved to the situations indicated by current guidelines and should not be conducted aggressively in preclinical HC. Blood donation, whenever possible, is a valid alternative for treating persons with HC.

## Short review on HC and blood donation

Because bloodletting has been the mainstay of treatment for HC for many decades (25), blood donation represents a valuable alternative to discarding the blood drown with therapeutic phlebotomy, with obvious advantages for both the patient /donor and for the blood collection center. However, blood donation for HC has been, and still is, a much-discussed issue. The two main concerns are whether the blood of donors with HC is safe for recipients of transfusions and whether these donations can be considered voluntary. Some potential safety issues (e.g., a possible increased infectious risk) were disproved by some studies but much more by the large amount of real-world data from centers that have being accepting HC blood donors for years. Other aspects remain unclear because of the paucity of specific data (e.g., the impact of elevated iron values on the biologic quality of RBC), and other concerns are still anchored in the culture of many national and local blood collection centers (e.g., ethical aspects of a non-altruistic donation). Moreover, blood collection facilities that would accept HC carriers as blood donors are confronted with financial and operational problems that may not be overcome easily and would require the support of clearly defined regulations that currently are not in place in every country.

### The blood donor with HC

Although included in the list of the rare disease (defined as a condition having a prevalence of <1 to 2,000), HC is not uncommon in European populations and is more frequent in those of prevalent Celtic ancestry. For instance, the homozygous HFE p.C282Y variant is present in about 10% of the population in Ireland, in 8% in the United Kingdom, in over 7% in Norway and France, and is not rare also in North Portugal (in 8 of 2,000 individuals) (26, 27). Thus, in these regions and in populations of European descent living in other continents, such as North America, South Africa and Australia, many undiagnosed people with HC are already donating blood.

Today, most cases of HC are detected upon accidental demonstration of elevated ferritin in the absence of symptoms of iron overload during a routine medical check or through ferritin measurement in blood donors. Middle-aged males represent the typical subjects diagnosed with HC who are referred for blood donation or who donate blood already (18). Not uncommonly, these individuals display risks for metabolic liver diseases (overweight, elevated blood pressure, dyslipidemia, regular alcohol intake), being these conditions frequent in the general population of Western countries. The predisposition to the development of fatty liver and the increased iron absorption in the duodenum caused by the underlying HFE genotype are often concomitant causes of increased ferritin, whereas ferritin elevation is usually moderate (less than 800–1,000 ng/mL). Because of their clinical and laboratory characteristics, persons with HC present to blood donation usually with normal to high-normal Hb levels, generally have lower rates of immediate complications compared to younger donors, and are more likely to be retained for blood donation in the long term, thus having a lower risk for transfusion-transmitted infections compared to first-time donors (28, 29). However, despite adequate Hb values and elevated ferritin, carriers of HC are not less susceptible to blood donation-related iron deficiency, as showed in studies that have examined the relationship between HFE genotype, iron homeostasis and the prevalence of iron deficiency and anemia. An analysis of over 23,600 Caucasian adults demonstrated that among subjects carrying one or two HFE variants anemia did occur as in those with wild-type genotype in the course of repetitive blood donation (30), and in a longitudinal evaluation similar changes of both Hb and iron status were described during regular blood donation in persons with or without HFE variants (31). It is therefore important to monitor blood donors with HC or other HFE variants for iron deficiency and anemia as all other donors, because of the potential impact of these side effects on general health, quality of life and long-term commitment to blood donation.

### Regulations on blood donation for HC

Both current European guidelines (15) and the Food and Drug Administration (FDA) permit the collection of blood from subjects with preclinical HC for manufacturing blood products for transfusion (https://www.accessdata.fda.gov/scripts/cdrh/cfdocs/cfcfr/CFRSearch.cfm?fr=630.15, accessed on 12/28/2023). In some European countries, regulations for accepting blood from eligible individuals with uncomplicated HC were implemented several years ago (e.g., the NHS Blood and Transplant in England and North Wales in 2001, Switzerland in 2004, Ireland even earlier) (32). In other countries (e.g., Germany), ongoing evaluations are considering to take a further step and to offer free access to phlebotomy in blood donation centers to all people with HC, including symptomatic patients. This strategy was already adopted in the United States, where a FDA variance enables blood center not only to collect blood from eligible persons with HC but to provide free phlebotomy to all persons presenting with HC regardless of eligibility for blood donation (16, 33). The financial implications of this policy were analyzed in a study from the New York Blood Center, that described a cost benefit to the center deriving from the blood units transfused, even considering those that had to be discarded because of eligibility issues (34). Overall, these and other experiences demonstrate an advantage for the blood centers that identify and treat healthy donors with HC with no financial loss to both the blood facility and the patient/donor (16, 35).

### Reasons for not allowing blood donation for HC

But then why are people with HC not universally accepted for blood donation? On one hand, reasons include concerns regarding a possible increased infectious risk related to HC donors, a lesser biologic quality of blood products, ethical issues, but also financial and operational aspects. On the other hand, national and local regulations on this topic are generally not evidence based and often not clear, leaving the elaboration of internal policies to the single centers.

As to increased infectious risks, three publications reported specifically on a low incidence of transfusion–transmissible infections from blood products obtained from persons with HC, although the results were based on small test subject numbers and in part conducted without a control group (16, 36, 37). Conversely, two other series described a higher incidence of bacterial contamination and a higher probability of concurrent viral infection (38, 39). Finally, a systematic review, that also included the observational studies mentioned above, found no evidence that blood from patients with uncomplicated HC is unsafe if iron levels are normalized (29). An analysis of over 50′000 blood donors in the United States found no statistically significant difference in positive routine infection screening tests (including syphilis, hepatitis B, hepatitis C, HIV) between donors with and without HC (17), and no seroconversion for transfusion-transmissible agents was reported in recipients of 1,402 serial donations in the first 27 months following the implementation of a program of comprehensive management of persons with HC and other HFE variants (16).

An impaired quality of red blood cells of HC blood donors is a potential consequence of the exposition to reactive iron forms. Even in preclinical HC, the enhanced iron absorption frequently exceeds the binding capacity of transferrin, the physiologic iron transporter, resulting in elevated transferrin saturation and in the appearance of redox-active non-transferrin bound iron in the circulation (19). An oxidative damage on cells due to free iron has been observed *in vitro* (40), but the amount of free iron in blood collections from donors with HC has actually been shown to be minimal (41). Also, blood from HC donors exhibits similar rates of hemolysis during storage as that from unaffected donors (42). However, although no real concerns exist regarding standard quality of red blood cell units, current evidence is insufficient for excluding that increased iron has a negative effect on the biologic and metabolic features of red cells of HC individuals, possibly accelerating the cellular changes normally occurring during storage of blood units. Overall-, studies on these aspects included limited numbers of red blood cell units and did not deliver conclusive results. In blood units drawn form 19 HC patients, a significantly decreased deformability and increased shear stress were observed compared to controls, but there was no clear evidence that an acute exposure to elevated iron levels was the cause of the observed differences (43). An Australian study on 15 volunteers with HC and 11 controls confirmed these data, suggesting that blood products from HC individuals can be more prone to damage and that this can be exacerbated in situations of elevated shear stress (e.g., transfusion during surgery) (44). However, the same authors also reported on an improvement of the mechanical properties of red blood cells of HC individuals following iron removal treatment, indicating a possibly crucial role of elevated ferritin in plasma in determining the previously observed cellular changes (45). Currently, a study ongoing in Switzerland aims to compare standard quality and biologic characteristics of red blood cell products of individuals with HC and of persons with reactively elevated ferritin on a larger number of blood units (registered as NCT05742035 at https://clinicaltrials.gov).

Finally, ethical issues are still perceived as a major obstacle to the acceptance of HC blood donors in many centers. This aspect has been confuted in one single specific publication (46) and by several transfusion medicine specialists in editorials and reviews (32, 42, 47) as well. The incentive to seek free care in a blood center has been traditionally viewed as a risk for not disclosing infectious risks, such as risky sexual behavior. However, as discussed above, this potential risk was not proven to hold true. Discussing the lack of an altruistic motivation if blood donation is performed as a no-cost therapeutic measure, Penning points out that persons with HC are basically free to decide whether their blood should be used for transfusion or should be rather discarded, and that they do not receive any reward if deciding to donate the blood. For these reasons, persons with HC can be considered as being altruistic volunteers, if they choose to become blood donors (46).

Centers accepting carriers of HC as blood donors should implement a policy of collaboration with the clinicians who refer these persons in order to ensure adequate medical surveillance (e.g., regular assessment of liver enzymes and correction of factors possibly impairing liver function) and should be involved in the clinical management of those persons who do not qualify for blood donation any longer but require regular iron depletion. Such programs may be not easy to implement in facilities with reduced medical staff or lacking physicians with specific knowledge. In addition, blood donation institutions may be not willing or able to afford process changes or implement financially demanding measures such as regular complete blood count and ferritin determinations. These aspects explain partly the variability of regional policies that can be found within the same country. Addressing operational and economic obstacles would require the support of national blood systems that, however, also deal very differently with the issue of HC and blood donation.

### Real-life practices of blood donation for HC

In 2013, the International Society of Blood Transfusion (ISBT) conducted the first, and to date only, survey on policies regarding the eligibility for blood donation of carriers with a HFE variant and/or patients with HC and collected information from 35 blood services around the world (14). A wide variation in practice, even within Europe, was observed. An interesting finding was that permissive donation policies were more frequent in countries with a high HC prevalence (e.g., United Kingdom, North America, South Africa, and Australia), while the majority of countries that did not respond to the survey or reported no acceptance of HC blood donors were those with low frequencies of HC (e.g., Belgium, Latvia, Romania, Slovenia, Hong Kong). At the time of the publication, asymptomatic HC carriers were accepted in 69% of the surveyed blood centers, and in nine of these facilities also symptomatic HC patients were allowed to donate blood, if eligible. Policies varied sometimes also among blood centers within a country, like in the United States, where centers require approval for collecting HC blood according to the FDA variance, as said above. According to the responses of the surveyed blood centers, the basis for accepting or rejecting HC subjects as blood donors relied on expert consensus, personal opinions or preference, internal regulations and, to a lesser extent, national legislation, revealing a clear a need for evidence-based policies. During the ten years following the publication of the ISBT survey, it is likely that more countries have changed they regulations and now allow blood donation of HC subjects. As to the contribution of preclinical or symptomatic HC donors to the total blood pool, the surveyed centers that were able to provide an estimate indicated a proportion of less than 5%. However, as the authors commented, the actual contribution to the blood supply was likely underestimated given the probably high number of active blood donors not being aware of having HC.

A program for comprehensive management of HC subjects was initiated in 2001 at the Department of Transfusion Medicine, Bethesda, Maryland (16) using both conventional whole blood donation and double-dose erythrapheresis as treatment. In that experience, with 76% of the 130 enrolled subjects meeting eligibility criteria for blood donation and 55% having donated blood previously, a total of 1,677 red blood cell units were collected, including 1,120 units suitable for transfusion, 385 units distributed for research use, and 172 units discarded in the first 27 months of the program. It was also showed that HC donors experienced personal satisfaction and benefitted from the quality and consistency of care provided at the blood center, and, at the same time, the blood center and the community took advantage from a substantial increase in the availability of RBC units for transfusion. These results were in line with similar previous experiences (17, 48, 49).

### Conclusions and practical suggestions for clinicians

As to date, no robust evidence undermines the refusal of persons with HC for blood donation. Evidence-based practices and harmonization of regulations are clearly required in order to offer the same therapeutic options to every individual with HC. According to the opinion of many transfusion specialists, the real ethic issues to be addressed concern the inequality of acceptance of these people in different blood institutions and the refusal of utilizing a willing but underutilized resource of blood products that could significantly contribute to the blood supply. And this despite the progressive shrinking of active blood donor pools ongoing in most countries. As stated by West and Eder, blood centers that did not implement HC donor programs should “examine why they do not and rectify this deficiency” (47).

At the conclusion of the short review, some practical, experience-based suggestions are provided for clinicians who consider referring patient with HC for blood donation.

- The patient with HC should not suffer of a major clinical condition (e.g., severe heart disease or vasculopathy) that poses an elevated risk for complications of phlebotomy or apheresis or even precludes performing these procedures.
- Although “healthy,” the patient with preclinical HC may not be eligible for blood donation. The evaluation of eligibility requires specific knowledge and should be discussed in advance with the blood donation facility.
- A medical documentation including a list of relevant diagnoses, available recent laboratory data and the results of the genetic testing for HFE should be provided to the blood donation center.
- Regular clinical and laboratory surveillance should be ensured and should not be delegated to the blood donation center, where the possibility of medical management and laboratory testing may be limited.
- The blood center should provide regular report on the ongoing treatment (e.g., number and frequency of procedures, relevant adverse events, compliance).

#### Scope statement

We present the results of a study of iron removal in preclinical hemochromatosis (HC) and in asymptomatic individuals with other HFE genotypes as performed in the highly regulated setting of blood donation. The scope of the study was to analyze the impact of frequent treatment procedures on healthy persons discovered to carry HFE variants and having elevated ferritin. Such individuals are frequently referred to blood donation centers, although the indication for treatment does not always comply with current published guidelines. An intensive iron removal treatment results in potentially harmful side effects also in otherwise healthy individuals. This aspect must be considered in the clinical management of persons with preclinical HC or with other conditions where iron removal is indicated. Our data point out that unnecessary iron removal in asymptomatic individuals should be avoided and that, when indicated, a less aggressive treatment should be the choice in subjects with moderately elevated ferritin and no symptoms of iron overload. A further aim of the present article is to provide clinicians with information on the advantages and limits of blood donation as a therapeutic option, and to illustrate the complexity of the issue of offering this service in a blood donation facility.

## Data availability statement

The raw data supporting the conclusions of this article will be made available by the authors, without undue reservation.

## Ethics statement

The studies involving humans were approved by Ethikkommission Nordwest und Zentralschweiz. The studies were conducted in accordance with the local legislation and institutional requirements. The participants provided their written informed consent to participate in this study.

## Author contributions

LI: Conceptualization, Data curation, Formal analysis, Funding acquisition, Investigation, Methodology, Project administration, Resources, Supervision, Validation, Writing – original draft, Writing – review & editing. GL: Investigation, Writing – review & editing. MM: Formal analysis, Writing – review & editing. VP: Investigation, Writing – review & editing. PB: Formal analysis, Methodology, Validation, Writing – review & editing. MC: Data curation, Formal analysis, Methodology, Writing – review & editing. AH: Conceptualization, Writing – review & editing. JP: Conceptualization, Writing – review & editing. NW: Conceptualization, Writing – review & editing. AB: Conceptualization, Funding acquisition, Resources, Writing – review & editing.
